# Anti-Glycoprotein G Antibodies of Herpes Simplex Virus 2 Contribute to Complete Protection after Vaccination in Mice and Induce Antibody-Dependent Cellular Cytotoxicity and Complement-Mediated Cytolysis

**DOI:** 10.3390/v6114358

**Published:** 2014-11-12

**Authors:** Staffan Görander, Maria Ekblad, Tomas Bergström, Jan-Åke Liljeqvist

**Affiliations:** Department of Infectious Diseases, Section of Virology, Guldhedsgatan 10 B, S-413 46 Gothenburg, Institute of Biomedicine, The Sahlgrenska Academy, University of Gothenburg, Box 100, 405 30 Göteborg, Sweden; E-Mails: staffan.gorander@gu.se (S.G.); maria.ekblad@microbio.gu.se (M.E.); tomas.bergstrom@microbio.gu.se (T.B.)

**Keywords:** glycoprotein G, herpes simplex virus 2, vaccination, antibodies

## Abstract

We investigated the role of antibodies against the mature portion of glycoprotein G (mgG-2) of herpes simplex virus 2 (HSV-2) in protective immunity after vaccination. Mice were immunized intramuscularly with mgG-2 and oligodeoxynucleotides containing two CpG motifs plus alum as adjuvant. All C57BL/6 mice survived and presented no genital or systemic disease. High levels of immunoglobulin G subclass 1 (IgG1) and IgG2 antibodies were detected and re-stimulated splenic CD4^+^ T cells proliferated and produced IFN-γ. None of the sera from immunized mice exhibited neutralization, while all sera exerted antibody-dependent cellular cytotoxicity (ADCC) and complement-mediated cytolysis (ACMC) activity. Passive transfer of anti-mgG-2 monoclonal antibodies, or immune serum, to naive C57BL/6 mice did not limit disease progression. Immunized B‑cell KO mice presented lower survival rate and higher vaginal viral titers, as compared with vaccinated B-cell KO mice after passive transfer of immune serum and vaccinated C57BL/6 mice. Sera from mice that were vaccinated subcutaneously and intranasally with mgG-2 presented significantly lower titers of IgG antibodies and lower ADCC and ACMC activity. We conclude that anti-mgG-2 antibodies were of importance to limit genital HSV‑2 infection. ADCC and ACMC activity are potentially important mechanisms in protective immunity, and could tentatively be evaluated in future animal vaccine studies and in clinical trials.

## 1. Introduction

Herpes simplex virus 2 (HSV-2) infects the genital mucosa and establishes a life-long infection in dorsal root sensory ganglia (DRG). After primary infection HSV-2 reactivates and may cause recurrent and painful genital ulcerations, or more commonly asymptomatic shedding. According to estimations of World Health Organization more than 500 million individuals are infected globally [1]. Moreover, HSV-2 infection is associated with a significant increased risk of HIV acquisition. Different vaccine candidates have been tested, using the HSV-2 glycoprotein B (gB-2), and/or glycoprotein D (gD-2) as antigens. Despite promising data from animal studies, results in vaccine clinical trials have been discouraging with limited or no protection against HSV-2 infection or disease [2,3,4,5]. These failures stimulate the development of new vaccine candidates. 

The envelope glycoprotein G (gG-2) protein is cleaved into one secreted moiety (sgG-2), and one portion anchored to the cell-membrane and the virion (mgG-2). The latter portion is matured by heavy *O*-glycosylation in mammalian cells and contains several clusters of single N-acetyl galactosamine with affinity for *Helix pomatia* (HPA) lectin. Both gG-2 proteins elicit exclusively type-specific antibody responses in humans [6,7,8,9,10], and mgG-2 is commonly used as antigen for serodiagnosis of HSV-2 infection in clinical settings. 

We showed earlier that subcutaneous (sc) and intranasal (in) immunization with a prophylactic vaccine based on mgG-2 and CpG induced partial protection (73% survival) in a mouse genital model [11], and that protection was associated with the IFN-γ production by CD4^+^ T cells. A novel finding was that a majority of immune sera exerted antibody-dependent cellular cytotoxicity (ADCC) and antibody-dependent complement-mediated cytolysis (ACMC) activity, but no neutralization (NT) activity. Although no obvious correlations between NT activity and protection are described in animal studies [12,13], and vaccine clinical trials [4,5], NT activity has often been used as a marker of the antibody responses. In a recent follow-up report from GSKs clinical trial of HSV negative women (Herpevac Trial), vaccinated with gD-2, 82% protection was observed against HSV-1 genital disease. Interestingly, the antibody levels against gD-2, but not the cellular immune responses, correlated to immune protection [14]. Furthermore, neutralizing antibody titers were significantly higher to HSV-1 than to HSV-2, an observation that may explain the failure to protect against HSV-2 infection [15]. These data justify studying the role of antibodies in protection in other vaccine candidates. We here evaluated the role of anti-mgG-2 antibodies in the clearance of genital HSV-2 infection, and for induction of protective immunity after vaccination. For this purposes B-cell deficient mice and passive transfer of antibodies were used. We also evaluated whether the immunization protocol modified IgG titers and ADCC and ACMC activity by comparing intramuscular with sc plus in administrations. 

## 2. Materials and Methods

### 2.1. Ethics Statement

This study was carried out in accordance to the rules stated by the Swedish board of agriculture. All animal experiments were approved by the ethical board in Gothenburg (Dnr 171-2013).

### 2.2. Mice 

Six- to eight-week-old female C57BL/6 mice (Harlan Laboratories, Inc, Indianapolis, IN, USA) and B-cell knock-out (KO) mice, B6.129S2-*Ighm^tm1Cgn^*/J, (µMT) [16] (The Jackson Laboratory Bar Harbor, ME, USA) were used in all experiments. Isoflurane (Baxter, Kista, Sweden) was used as anesthesia.

### 2.3. Cells and Viruses

African green monkey kidney (GMK-AH1) cells were cultured in Eagle's minimal essential medium supplemented with 2% calf serum and antibiotics. Baby hamster kidney (BHK21) cells were propagated in Glasgow minimum essential medium with 8% calf serum, 1% L-glutamine, and 8% tryptose phosphate broth. Wild-type HSV-2 strain 333 was used as challenge virus.

### 2.4. The mgG-2 Protein

The mgG-2 protein used for immunization was produced by HPA lectin affinity chromatography [17,18]. The antigen was characterized recently and showed high purity, lack of other cross-reactive HSV-2 proteins, as determined by Western blot and by ELISA using HSV-1 positive sera, no cell toxicity or endotoxin contamination [11].

### 2.5. Monoclonal Antibodies (MAbs) and Immune Sera

Four anti-mgG-2 IgG1 MAbs (O1C5B2, O2B4A6, O3G11H7 and O1B9E5) were used [18]. Immune sera were collected and pooled from ten C57BL/6 mice immunized intramuscularly (im) × 3 with mgG-2 and CpG plus alum. The concentration of mgG-2 specific IgG antibodies was analyzed in an ELISA using a standard curve created by a mixture of the MAbs. 

### 2.6. Adjuvant

Synthetic oligodeoxynucleotides containing two optimal mouse CpG motifs (ODN1826, TCC ATG ACG TTC CTG ACG TT; Operon Biotechnologies GmbH, Cologne, Germany) and alum (AL hydrogel “85” 2%, Brenntag Nordic A/S, Ballerup, Denmark), were used as adjuvant.

### 2.7. Immunization and Challenge

C57BL/6 and µMT mice, on C57BL/6 background, were immunized by administrating three im injections in the caudal thigh muscle with 10-days interval. Each dose contained 2.5 µg mgG-2, 20 µg CpG and 250 µg alum diluted in Tris-buffered saline (TBS). One control group was immunized im with mgG-2 and alum, and one control group with CpG and alum alone. In parallel, 10 C57BL/6 mice were immunized with mgG-2 and CpG with one sc and two in administrations as described [11]. Three weeks after the third immunization these animals were euthanized and 1.5 mL blood was collected for analyses of ADCC, ACMC and NT activity as well as anti-mgG-2 IgG antibodies. 

Six days before challenge the mice were injected sc with 3 mg of medroxiprogesteron (Depo-Provera, Pfizer AB, Sollentuna, Sweden). Mice were challenged intravaginally (i.vag.) with 1 × 10^5^ (25 × 50% lethal dose) plaque forming units (PFU) of HSV-2. Viral replication in vagina was measured by collecting vaginal washes at day 3 post infection (p.i.) and infectious HSV-2 was detected by a plaque assay as described [11]. Vaginal inflammation and disease were graded as follows: healthy (score, 0), genital erythema (score, 1), moderate genital inflammation with blisters (score, 2), severe and purulent genital lesions with loss of hair (score, 3), and hind-limb paralysis and/or general bad condition (score, 4).

### 2.8. Serum Samples and ELISA

Serum was collected 14 days after the third immunization. IgG antibodies against mgG-2 were detected by an indirect ELISA as previously described [9,11]. Anti-mouse subclass-specific peroxidase-conjugated IgG were used as conjugates (Southern Biotech, Birmingham, AL, USA) at a 1:1000 dilution. The antibody titer was defined as the reciprocal value of the highest serum dilution giving an optical density (OD) greater than a negative serum plus 0.2 OD units. 

### 2.9. CD4^+^ T Cell Proliferation Assay and Cytokine Detection

The assay is described in detail elsewhere [11]. Briefly, spleens from two mice per group were pooled. The experiment was performed twice for each group. Non-immunized C57BL/6 mice were used as controls. CD4^+^ T cells were purified using the BD IMag™ Mouse CD4^+^ T Lymphocyte Enrichment Set (BD Bioscience, Stockholm, Sweden). Cells were incubated with 3 µg/mL of mgG-2. Concanavalin A, at a concentration of 2.5 µg/mL, was used as positive control. Cells were labeled with 1 µCi of [6-3H^3^]thymidine (Amersham Biosciences, Piscataway, NJ, USA), and incubated overnight. Cell proliferation was expressed as mean stimulation index (SI), defined as the amount of [6-3H^3^]thymidine incorporated into antigen-stimulated cultures divided by the mean amount incorporated into corresponding unstimulated control cultures. Samples presenting SI > 3 were considered as positive. Supernatants from the cell proliferation assay, collected at 96 h, and vaginal washes collected at day 1 p.i., were analyzed in a cytokine ELISA for IFN-γ (Duoset R&D kit, Minneapolis, MN, USA).

### 2.10. Passive Transfer of Immune Serum to Immunized µMT Mice 

Immunized µMT mice were injected intraperitoneally (ip) 48 h before challenge with 200 µL immune serum collected from previously immunized C57BL/6 mice. Each mouse received 300 µg of mgG-2 specific IgG antibodies. 

### 2.11. Passive Transfer of MAbs or Immune Serum to Naive Mice 48 h before Challenge

In the first passive transfer experiment, 200 µg of MAbs O1C5B2, O3G11H7, O2B4A6, or O1B9E5, were injected ip followed by i.vag. challenge with 1 × 10^4^ PFU of HSV-2. In the second passive transfer experiment, 200 µg of the MAb O2B4A6 or O1B9E5, were given ip followed by challenge with 1 × 10^5^ PFU HSV-2. In the third passive transfer experiment, 500 µg of the MAb O2B4A6 or O1B9E5, were given ip followed by challenge with 1 × 10^5^ PFU of HSV-2. In the last passive transfer experiment immune serum was given as described above.

### 2.12. NT Assay

The neutralization capacity of immune serum was tested in a plaque reduction assay. One hundred PFU of HSV-2 was mixed with serum at a 1:20 start dilution. Human serum from an HSV negative subject was used as complement source at a 1:20 dilution. The mixture of virus and serum was transferred to monolayers of GMK-AH1 cells. The NT titer was defined as the reciprocal value of the highest serum dilution giving 50% plaque reduction as compared with controls. An HSV-2-positive human serum was used as a positive control.

### 2.13. Antibody Dependent Cellular Cytotoxicity (ADCC) and Complement-Mediated Cytolysis (ACMC)

ACMC was performed as described while for the ADCC assay we used a slightly modified method from what have previously been described [11]. Briefly, monolayers of BHK21 target cells were infected with HSV-2, at a multiplicity of infection of 4, and labeled with 100 µCi Na_2_^51^CrO_4_/million cells (Perkin Elmer, Waltham, MA, USA). As effector cells we used fresh peritoneal macrophages from C57BL/6 mice [19]. After preparation macrophages were re-suspended in Iscoves with 5% inactivated foetal calf-serum, and 1% antibiotics. Target cells (4 × 10^3^)/well and serum samples, at a 1:20 dilution, were added to a 96-well plate (Nuclon Delta Surface, Thermo Fischer Scientific, Roskilde, Denmark). Effector cells were added, followed by incubation for 18 h at 37 °C in a humid atmosphere of 5% CO_2_. An effector/target cell ratio of 100:1 was used. Supernatants were collected by harvesting frames (Molecular Devices Sunnyvale, CA, USA) and counted using a Cobra Auto-gamma counter (model 5002/5003; Packard Instrument Company, Meriden, CT, USA). The percentage of specific release of ^51^Cr was calculated by the following formula: 

$$\frac{\left(counts\;per\;minute\;of\;immune\;serum-counts\;per\;minute\;of\;nonimmune\;serum\right)\;}{\left(counts\;per\;minute\;of\;maximum\;release-counts\;per\;minute\;of\;nonimmune\;serum\right)}\;\times \;100$$

### 2.14. Statistics

SigmaPlot 12 software [20] for Microsoft Windows was used for statistical calculations. Fisher’s exact test was used for survival data, ADCC and ACMC. One-way ANOVA on ranks was used for IFN-γ values, IgG titer and HSV-2 viral titer (PFU). P values of <0.05 were considered statistically significant.

## 3. Results 

### 3.1. Detection of Antibodies in Vaccinated Mice

**Figure 1:**
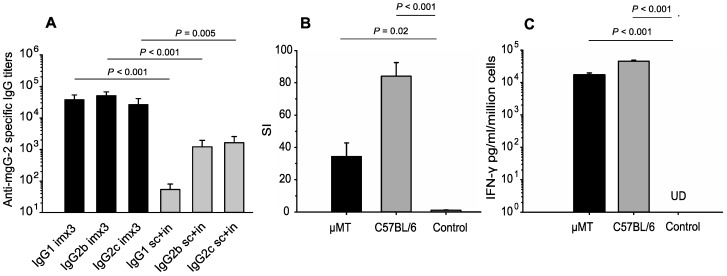
B- and T-cell responses in C57BL/6 and µMT immunized mice. Mice were immunized with three doses of mgG-2 and CpG plus alum intramuscularly (im × 3), (*n* = 19), or with mgG-2 and CpG with one subcutaneous dose followed by two intranasal doses (sc plus in), (*n* = 10). (**A**) Serum was subjected to an mgG-2 specific ELISA for detection of IgG subclass antibodies. (**B**) Enriched CD4^+^ splenic T cells were stimulated with 3 µg/mL of mgG-2 for 4 days. Proliferation was measured by radiolabeled thymidine incorporation and presented as SI units. (**C**) IFN-γ production in the supernatants of stimulated cells was measured with an ELISA with a detection limit of 10 pg/mL. Error bars represent + SEM. UD, undetectable.

We analyzed the anti-IgG responses after vaccination with mgG-2 and adjuvant and compared the IgG1, IgG2b and IgG2c titers after im immunizations with sc plus in immunizations. Sera were collected 14 days after the third immunization of vaccinated mice. Intramuscularly immunized C57BL/6 mice showed robust antibody responses against mgG-2. The mean IgG1, IgG2b and IgG2c end-point titer was 3.7 × 10^4^, 5.1 × 10^4^ and 2.6 × 10^4^, respectively, which was significantly higher than for mice immunized sc plus in (IgG1 54, *P <* 0.001; IgG2b 1.2 × 10^3^, *P* < 0.001; IgG2c 1.6 × 10^3^, *P* = 0.005), (Figure 1A). We conclude that im immunized mice using CpG and alum as adjuvant induced significantly higher IgG antibody titers as compared with sc plus in immunization with CpG as adjuvant. As expected, sera from vaccinated µMT mice were devoid of IgG1 and IgG2b/c antibodies (data not shown). 

### 3.2. CD4^+^ T-Cell Responses Against mgG-2 and IFN-γ Production 

Three weeks after the third im immunization enriched splenic CD4^+^ T cells from both C57BL/6 and µMT vaccinated mice were collected and re-stimulated with mgG-2 antigen for 96 h. Proliferation of cells and production of IFN-γ in the supernatant from the cell culture proliferation assay were measured. As shown in Figure 1B, CD4^+^ T cells from both mouse strains showed significantly higher SI (C57BL/6 = 84, µMT = 34) as well as higher IFN-γ levels as compared with controls (Figure 1C). 

### 3.3. Survival and Disease Score in Vaccinated C57BL/6 Mice

**Figure 2:**
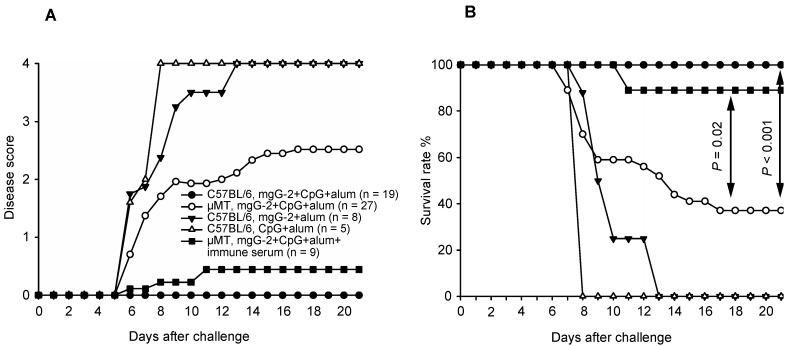
(**A**) Disease score and (**B**) survival rate of mgG-2 immunized C57BL/6 and µMT mice after vaginal challenge with 1 × 10^5^ PFU (25 × 50% lethal dose) of HSV-2, (day 0). Mice were immunized three times intramuscularly with mgG-2 and CpG plus alum (C57BL/6 and µMT), or with mgG-2 and alum, or with CpG and alum (C57BL/6). Immune serum (300 µg anti-mgG-2 antibodies) from vaccinated C57BL/6 mice was passively transferred to µMT mice 48 h before challenge.

We investigated whether anti-mgG-2 antibodies contribute to protection after vaccination. C57BL/6 mice, vaccinated im × 3 with mgG-2 and CpG plus alum, were fully protected against genital and systemic disease as well as death (Figure 2). Mice vaccinated with CpG and alum and mgG-2 and alum, all showed severe disease and succumbed after challenge before day 14 post infection. 

### 3.4. Survival and Disease Score in Vaccinated µMT Mice and Passive Transfer of Immune Serum

The mgG-2 vaccinated µMT mice presented significantly higher disease score (2.5) and a lower survival rate (37%, *P* < 0.001), as compared with fully protected C57BL/6 mice. After substitution with anti-mgG-2 immune serum 48 h before challenge, the disease score was reduced to 0.44 and survival rate was significantly improved to 89% (*P* = 0.02), (Figure 2). Thus, we show that anti-mgG-2 antibodies contribute to protection after vaccination with mgG-2.

### 3.5. Passive Transfer of Anti-mgG-2 MAbs or Immune Serum to Naive C57BL/6 Mice

**Figure 3:**
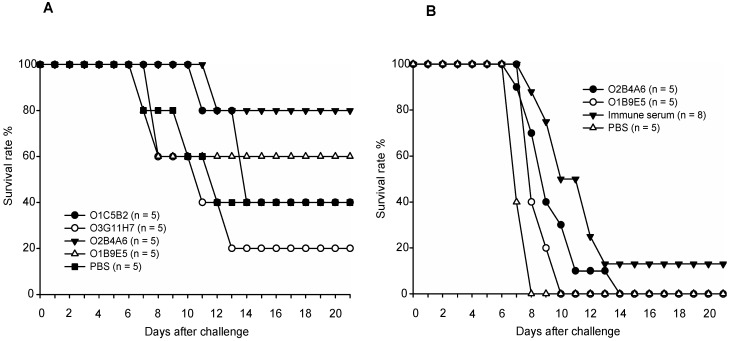
Survival rate of naive C57BL/6 mice after passive transfer of anti-mgG-2 MAbs or immune serum. (**A**) Mice received intraperitoneally 200 µg each of the MAbs O1C5B2, O2B4A6, O3G11H7 or O1B9E5 48 h before i.vag. challenge with 1 × 10^4^ PFU (2.5 × 50% lethal dose) of HSV-2. (**B**) The MAb O2B4A6 or O1B9E5 (200 µg/mouse), immune serum (300 µg mgG-2 antibodies), or non-immune serum (200 µL/mouse), were given intraperitoneally 48 h before i.vag. challenge with 1 × 10^5^ PFU (25 × 50% lethal dose) of HSV-2.

Four different anti-mgG-2 MAbs were injected separately (200 µg) ip 48 h before i.vag. challenge with 1 × 10^4^ PFU (2.5 × 50% lethal dose) of HSV-2. As the survival and disease score curves correlated well for each mouse the survival data are presented. The MAbs O2B4A6 and O1B9E5 conferred partial protection with a survival rate of 60% and 80%, respectively, (Figure 3A). As not all controls succumbed in the first experiment, 200 µg of these two MAbs were given separately using a higher challenge dose of HSV-2 (1 × 10^5^ PFU, 25 × 50% lethal dose) in a second experiment. Although a delay in disease progress and death was observed, all mice including controls succumbed (Figure 3B). Similar results were obtained when 500 µg of each of MAb O2B4A6 or O1B9E5 were given ip (data not shown). Finally, pooled immune serum (300 µg anti-mgG-2 antibodies) were injected ip 48 h before challenge with 1 × 10^5^ PFU of HSV-2. Only one of eight mice survived (Figure 3B). We conclude that neither anti-mgG-2 MAbs nor immune serum given before challenge had any significant effect on survival or development of genital or systemic disease in naive non-immunized mice. 

### 3.6. Viral Load and IFN-γ Response in Vaginal Washes

**Figure 4:**
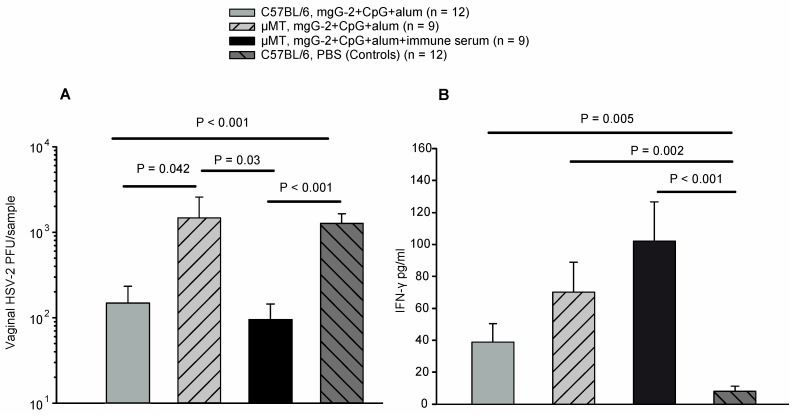
Viral load and IFN-γ concentration in vaginal washes of vaccinated C57BL/6 mice, vaccinated µMT mice with and without immune serum, and non-immunized C57BL/6 controls. (**A**) Infectious viral particles (PFU) were detected at day three post infection by titration on GMK-AH1 cells. The detection limit was 20 PFU/sample. (**B**) IFN-γ was detected by an ELISA in vaginal secretions at day 1 post infection. The detection limit was 10 pg/mL. The error bars represent + SEM.

Vaccinated C57BL/6 mice showed significantly reduced viral titers (mean 1.5 ×10^2^ PFU) as compared with vaccinated µMT mice (mean 1.5 × 10^3^ PFU), (*P =* 0.042), and with non-immunized C57BL/6 controls (mean 1.3 × 10^3^ PFU), (*P <* 0.001). Passive transfer of immune serum to vaccinated µMT mice reduced the viral titers significantly (mean 9.6 × 10^1^ PFU), as compared with those not given immune serum (*P* = 0.03), or controls (*P* < 0.001) (Figure 4A). Significantly higher levels of IFN-γ in vaginal secretions at day one p.i. were detected in immunized C57BL/6 mice (*P* = 0.005), in immunized µMT mice (*P* = 0.002), and in immunized µMT mice that received immune serum (*P* < 0.001), as compared with controls (Figure 4B). We conclude that anti-mgG-2 antibodies after vaccination significantly increased the production of IFN-γ and reduced the viral load in vaginal washes.

### 3.7. NT, ADCC and ACMC Activity in Sera from mgG-2 Vaccinated C57BL/6 Mice

We investigated if the immunization protocol could influence the functional activity of the mgG-2 antibodies. Immune sera were collected from C57BL/6 mice 14 days after the third immunization. First, sera were collected from im immunized mice using CpG and alum as adjuvant and, second, mice immunized with sc plus in administrations using CpG as adjuvant. Sera were analyzed with a plaque reduction assay for the presence of NT activity. Despite high IgG1 titers, none of the sera showed NT activity with or without complement (titer < 20) (Table 1). For 10 mice, using the im immunization protocol, sufficient volumes were available for the ADCC assay. All sera (10/10) showed ADCC activity (titer ≥ 20), while for 10 sera collected from mice with sc plus in immunization protocol, 4/10 presented ADCC activity, (*P* = 0.01). Nineteen sera from mice immunized im × three were available and showed all ACMC activity (titer ≥ 25), while 5/10 sera from mice immunized sc plus in presented ACMC activity (*P* = 0.002), (Table 1). Complement alone and immune serum alone were devoid of ACMC activity, and non-immune serum showed neither ADCC nor ACMC activity (titer < 1/20 and <1/25). 

**Table 1:** ADCC, ACMC and NT activity of sera from intramuscularly vaccinated C57BL/6 mice with mgG-2 and CpG plus alum, and from subcutaneously and intranasally immunized C57BL/6 mice with mgG-2 and CpG. Results shown are number of positive/total number tested.

Method	Immunization schedule	
	sc × 1 + in × 2	im × 3
ADCC, titer ≥20	4/10	10/10^a^
ACMC, titer ≥25	5/10	19/19^b^
NT^c^, titer ≥20	0/10	0/19

^a^ *P* = 0.01; ^b^ *P =* 0.002; ^c^ with and without complement.

## 4. Discussion 

We show that anti-mgG-2 antibodies, elicited after vaccination of mice with mgG-2 and adjuvant, are important for the outcome after genital challenge with HSV-2. When mice were immunized im × three with CpG and alum as adjuvant, complete protection from death and disease was observed. These adjuvants in combination with different subunit HSV-2 antigens given im have been used successfully to elicit robust B- and T-cell immune responses [21,22]. We also show that im immunization protocol using CpG and alum as adjuvant elicited significantly higher levels of IgG antibodies and higher ADCC and ACMC activity, as compared with sc plus in administrations using CpG as adjuvant. Thus, our data emphasize the importance of the adjuvant and the administration protocol used together with the mgG-2 antigen. 

### 4.1. NT, ADCC and ACMC Activity of Anti-mgG-2 Antibodies

In the present study, as described in our earlier report [11], no NT activity in the presence or absence of complement was detected in immune sera. Different anti-mgG-2 MAbs and rabbit immune serum raised against purified mgG-2 also lack NT activity [11,12,23,24]. The results presented herein suggest that anti-mgG-2 antibodies are involved in protective immunity by other functional properties than NT activity. Few studies on ADCC have been performed in mice, probably due to difficulties in preparation of effector cells and weak responses. However, in a mouse model using passively transferred anti-HSV-2 MAbs (gB, gC, gD, gE and mgG-2), followed by a lethal footpad inoculation, a good correlation was observed between protection and ADCC but not with ACMC or NT activity [12].

The importance of ADCC and ACMC activity in protection after i.vag. HSV-2 infection is, to our knowledge, lacking. Chu *et al*. showed that an Fcγ-receptor dependent mechanism is involved in antibody dependent protection against genital HSV-2 infection [25]. Furthermore, low levels of antibodies capable of mediating ADCC, may have contributed to the low efficacy observed in clinical HSV-2 vaccine trials, using gD and/or gB as immunization antigens [26]. In this study, all sera from im immunized mice showed ADCC and ACMC activity, while approximately half of the sera collected from mice with immunization protocol using sc plus in administrations. As the mouse IgG2 subclass binds to Fcγ-RI more efficiently than IgG1 [27], the significantly higher titers of IgG2b and IgG2c antibodies detected for im immunized animals, could explain the increased ADCC and ACMC activity. However, we cannot exclude that the addition of alum as adjuvant in combination with im immunization route induce a different antibody profile that is more disposed to induce ADCC and ACMC activity. Further studies using Fcγ-receptor and complement depleted mice are warranted to investigate the biological relevance of ADCC and ACMC for protection against genital infection and disease in mgG-2 immunized animals.

### 4.2. B-Cell KO Mice 

Vaccinated B-cell KO mice presented significantly higher disease scores and lower survival rate as compared with immunized C57BL/6 mice (Figure 2). Passive transfer of immune serum to vaccinated µMT mice almost completely prevented death and disease as well as reducing the genital viral load to levels comparable with vaccinated C57BL/6 mice. These data suggest that B-cells, and presumably anti-mgG-2 antibodies, have a role in protective immunity after i.vag. challenge with a virulent HSV-2 strain. Other studies using µMT mice immunized with attenuated thymidine kinase negative HSV-2 [28,29], or replication-defective HSV-2 mutant [30], and passive transfer of immune serum [18,20], have described that antibodies limit but do not prevent genital infection early (≤ three days) after HSV-2 challenge with reduction of genital replication and genital and neurological disease.

CD4^+^ T cell proliferation (SI) was lower in the immunized µMT mice (SI = 34) as compared with immunized C57BL/6 mice (SI = 84). A relevant question is whether the µMT mice have an intact CD4^+^ T cell memory function [31]. The production of IFN-γ in the cell proliferation assay was similar in µMT mice as compared with immunized C57BL/6 mice (Figure 1C). The finding that IFN-γ was rapidly produced in the vagina of immunized µMT mice after challenge (Figure 4B), also suggests that a T cell memory was established. Functional T cell responses in µMT mice after i.vag. HSV-2 infection have also been described by others [28,29].

### 4.3. Passive Transfer of Antibodies to Naive Mice

Several studies have described that passive transfer of polyclonal immune serum to non-immunized mice or guinea-pigs does not prevent HSV-2 infection but reduces genital and neurological disease, and in the guinea-pig model also subsequent recurrent shedding [25,30,32,33]. Similar results have been described using passive transfer of MAbs directed against gB or gD followed by i.vag. challenge with HSV-1 [34,35] or HSV-2 [25]. However, passive transfer of anti-HSV-2 serum to naive mice was not so effective in preventing death and local replication in an ocular challenge model with HSV-2 [36]. Moreover, MAbs directed to gB, gC or gD administrated intravenously protected from systemic but not from i.vag. challenge with HSV-2 [37]. The discordant results may be explained by differences in the amount and specificity of antibodies given, virus dose, and the challenge model used. Results presented in this study suggest that anti-mgG-2 antibodies are protective in immunized mice most likely together with T cells but ineffective acting alone. 

### 4.4. Concluding Remarks

Data presented in this report suggest that mgG-2 has an important, but to date unknown function, in the genital infection, which can be targeted by immune responses elicited after vaccination. The results emphasize the importance of inducing antibodies after vaccination, and may result in a paradigm shift in the understanding of the correlate of protection [38]. Non-neutralizing antibodies which contribute to protection, possibly mediated by ADCC or other humoral effector functions, have been described not only for HIV, but also for other RNA and DNA viruses [39]. The generation of antibodies, which present ADCC and ACMC activity, may therefore be relevant in the development of vaccines against HSV-2 infection.
